# Progress in Surgery

**Published:** 1898-12-24

**Authors:** 


### PROGRESS IN SURGERY.

### TUMOURS.

(*Continued from page* 199.)

**Etiology of Tumours.**—This subject is one of the most important that surgeons and pathologists of the day have to grapple with. S. R. Park has written recently an interesting paper upon the subject. Dr. Park alludes to the etiological factors of sex, age, complexion, heredity and constitutional state. Sex has influence with regard to carcinoma only. Though females still perish in larger numbers than males from carcinoma the difference in numbers is less than it used to be. From 55 to 65 is the decade in which cancer is most often met with. The uterus and breast are attacked relatively early, having become *passé* with the conclusion of child-bearing life. There is a greater liability of brunettes than of blondes to suffer from cancer. Williams found the disease twice as frequent in brunettes. The heredity of cancer is a vexed question. A family history of the affection is found in 25 to 35 per cent. of the cases, but it is doubtful to what extent environment or infection from common sources account for this. The researches of Noel are alluded to, which go to show that a relation exists between arboreal cancers and those of man. There is not only the frequency of these tumours in habitations near woods, but a relatively large mortality among those persons whose occupation obliges them to work amid these surroundings. The work of the Italian scientists, Professor Sanfelice and Professor Roncali, and the remarkable results they have obtained with blastomycetæ obtained from cancers, are carefully described in this paper. The blastomycetæ are the fungi to which the common yeasts belong. They multiply by budding usually. They grow on acid media. The above workers found them within and around cancer cells, especially in the periphery of the tumours, and obtained cultures of them and produced tumours by the inoculation of these cultures. For example, the saccharomyces was injected into the mammary gland of a bitch, which lived for fourteen months, and then died with a definite tumour in the gland and with metastases in various organs. From the tumours thus produced, in some cases further cultivations were obtained and again inoculated.

Dr. Ed. Harrison[7] is the author of another paper on the subject of the etiology of growths, especially of the malignant type. Hausemann's views are described. That writer believes that each type of cell in the body has its own form of mitosis, and that in cancer the number and form of the mitosis is different from that found in the mother tissue. As there is rapid proliferation, the number of karyo-kinetic figures seen in a single field of the microscope is greatly increased, and the rate of growth can be fairly accurately estimated from the number of mitoses seen. Asymmetrical forms of mitosis, *i.e.*, mitosis in which an odd number of chromosomes are present, are common in cancer, whereas in normal animal cells the number of chromosomes is always even. In this paper of Harrison's the theory of "spermatic" influence of cells is enlarged upon, and also some recent work on glycogen as found in malignant tumours. Professor Maiers says of the theory of extension by "infection of the neighbourhood," that at first the multiplication of the epithelial cells by fission is the cause of the growth of the tumour, but later the developed cells are able to exert a contact influence on connective tissue cells. Creighton, of Cambridge, has been able to show sections taken from the margin of epithelial growths where, at a certain distance from the growth, the connective tissue cells have broken up into rounded groups of cells. Those still nearer the growth have an alveolar arrangement, and appear to be more spherical from multiplication and mutual pressure, and this alveolar meshwork containing cells is identical with that of the central parts of the tumour, which has been derived from pre-existing epithelial tissue. Creighton and others have also found that glycogen exists in many forms of tumour, as it does in the rapidly-growing structures of the fœtus. Harrison believes the cause of malignancy will only be explained when more is known of the cause of the development of fœtal organs, and says that the parasitic theory does not bear criticism.

Another paper on the etiology of tumours is that by Dr. Frank Hartley.[8] He quotes the authorities Maffuci and Sirleo, who deny any pathogenic properties existing in the blastomycetes of Sanfelice and others. They are led to believe, from personal observations, that only chronic inflammatory processes are caused by blastomycetæ, not carcinomata or sarcomata. Hartley points out that malignant tumours, even when there is no degeneration and ulceration, probably may develope substances (toxines), which act upon the tissues of the body and cause cachexia. For, independently of organisms, animal cells possess the power of producing chemical products which react upon the metabolism of tissue. Lipomata, multiple and symmetrical, as well as neuremata, multiple and symmetrical, when not congenital, are considered trophoneurotic.

**The Treatment of Inoperable Sarcoma and Carcinoma.** —Dr. William B. Coley[9] has published an account of the immediate and final results of the treatment of sarcomata with the mixed toxins of erysipelas and bacillus prodigiosus. The fluid used is the mixed toxins, sterilised by heating to 5 8 C., and unfiltered. Coley has not heard of any successful case treated with the erysipelas toxin alone. The toxins in some way, probably acting through the blood serum, cause a coagulation necrosis with fatty degeneration of tumour cells. The initial dose should be half a minim. Boiled water may be used to dilute the fluid and facilitate dosage. Each day the dose should be increased by one-half minim, until the reaction temperature reaches 102 deg. or 103 deg. Larger doses are borne if given subcutaneously remote from the tumour. If no beneficial results follow from three weeks' treatment, it is useless to continue it. In all Coley's successful cases marked improvement was seen within a week. One patient had the toxins steadily for two and a half years, and another for nearly four years, and no evil effects were produced. The macroscopical changes in the tumour first are marked decrease in vascularity, loss of the smooth, glossy appearance of the overlying skin, and later the presence of small wrinkles. The tumour becomes more mobile, and often marked decrease in size is noticed in three or four days. Pain is diminished soon. The injections undoubtedly cause increased susceptibility to infection from pathogenic germs, and therefore too great caution cannot be exercised in sterilising the skin and the needle. In the summary of final results Coley states that he has treated 140 cases, and, out of these, in 24 cases there was partial or complete disappearance of tumours; in 16 cases this was complete. Further, of these cases 8 were well 3 to 6 years; 9 were well 1½ to 3 years; 4 are well at present, 9 months to 1½ years; 3 died of recurrence from 7 months to 3½ years, after treatment. The spindle-celled sarcomata were most amenable to the treatment; in fact, of these more than half disappeared, and all showed marked improvement. A table is appended to the paper giving a list of 36 cases in which the treatment was used by other surgeons with partial or complete success.

M. C. Beretta[10] has collected no fewer than 73 cases in which injections of anti-cancerous serum have been used in the treatment of new growths. The serum is prepared by injecting repeatedly cancer juice into the horse, ass, or dog, and later obtaining the blood of these animals. No unquestionable case of cancer was cured, but the serum is said to be certainly useful in controlling pain, œdema, and hæmorrhage, and reducing the size of tumours and improving the aspect of ulcers. It has acted in this way in the most diverse forms of neoplastic growth. M. Beretta is not satisfied that its action is really specific, seeing that normal serum produces similar though less marked effects.

S. Fowler,[11] in a paper upon the toxin treatment of tumours, mentions six cases treated by Emmerich with serum obtained from a sheep which was dying from the effects of the injection of a virulent streptococcus culture. One recurrent carcinoma of the breast disappeared, but very little effect was produced upon the four other breast cases. Fowler also alludes to the use of the venom of the cobra capello. Cures of leprosy and elephantiasis are said to have followed inoculation with this poison, and it has long been known that the venom sometimes produces remarkable modifications in the nutrition of the parts bitten, such as the atrophy of a member. Repin used the dry venom, beginning with a dose of 1·40th of a milligram, and working up to 7 mgm. in a case of sarcoma of the tonsil. No alteration in the growth resulted.

**Oophorectomy for Inoperable Cancer of the Breast.**—Mr. Watson Cheyne[12] read a report at the Harveian Society upon two cases in which he had performed this operation. The first case was that of a woman, aged 34, with a recurrent carcinoma. There was an ulcer in the mid-axillary line; a hard mass, adherent to the chest wall, occupied almost the whole of the axilla; and there were numerous enlarged glands in the posterior triangle. Four and a half months after the operation the ulcer had shrunk in size, the mass in the axilla was distinctly smaller, softer, and freely movable, and the glands in the neck were smaller, and some had entirely disappeared. Three months later, however, there was again distinct increase in the size of the growth, and reappearance of the large glands. The second case, a woman of 33, had carcinoma of both breasts. There was thought to be slight improvement three weeks after the operation, but this was not maintained. Internal deposits appeared, and she died five months later. Mr. Cheyne held it clear that there is a distinct connexion between the normal epithelium of the breast and the ovaries, and also between cancerous epithelium of the breast and the ovaries. Unfortunately in all cases the effect of the removal of ovarian influence has been only transitory, whilst in many cases no effect has been produced.

Mr. Harold Stiles, in the discussion which followed the reading of the paper mentioned, alluded to Dr. Beatson's first case, which he had seen recently. Though in that case there had been complete disappearance of the disease for several months, recurrence had now become manifest. Other surgeons at the Edinburgh Royal Infirmary had tried the treatment with the result that in some cases the disease had been temporarily arrested, whilst in others it had been uninfluenced. Dr. Beatson considers that thyroid treatment is necessary if the patient be near the menopause, and that it is useless if metastatic growths have developed. Dr. Doran alluded to the difficulty often met with in removing every particle of ovarian tissue. The ovarian ligament often contained ovarian tissue. Cheyne thinks it would be well to remove as much of the noticeable disease as possible at the same time, or possibly later, for it would clearly be of advantage that there should be as little cancerous epithelium as possible in which to produce atrophy. Mr. Boyd had done this in one case, but it was too early to speak of the result.

[7] Quarterly Med. Jour., July, 1898. [8] Annals of Surgery, April, 1898. [9] Jour. of the Amer. Med. Assoc., Aug., 1898. [10] Lancet, Aug., 1898. [11] Amer. Jour. Med. Sci., April, 1898. [12] Lancet, April, 1898.

